# Experimental and Statistical Study on the Properties of Basic Oxygen Furnace Slag and Ground Granulated Blast Furnace Slag Based Alkali-Activated Mortar

**DOI:** 10.3390/ma16062357

**Published:** 2023-03-15

**Authors:** Hakan Özkan, Nausad Miyan, Nihat Kabay, Tarik Omur

**Affiliations:** 1 Oyak Cement Concrete Paper Group/Betâo Liz SA, 1099-020 Lisbon, Portugal; 2 Department of Civil Engineering, Yildiz Technical University, Istanbul 34220, Turkey; 3 LBA Design and Consultancy, Istanbul 34750, Turkey

**Keywords:** basic oxygen furnace slag, alkali-activated mortar, response surface methodology, statistical analysis, sodium hydroxide, optimization

## Abstract

Basic oxygen furnace slag (BOFS) is a waste material generated during the steelmaking process and has the potential to harm both the environment and living organisms when disposed of in a landfill. However, the cementitious properties of BOFS might help in utilizing this waste as an alternative material in alkali-activated systems. Therefore, in this study, BOFS and blast furnace slag were activated with varying dosages of NaOH, and the fresh, physical, mechanical, and microstructural properties were determined along with statistical analysis to reach the optimal mix design. The test results showed that an increase in BOFS content decreased compressive and flexural strengths, whereas it slightly increased the water absorption and permeable pores of the tested mortar samples. On the contrary, the increase in NaOH molarity resulted in a denser microstructure, reduced water absorption and permeable pores, and improved mechanical properties. Statistically significant relationships were obtained through response surface methodology with optimal mix proportions, namely, (i) 24.61% BOFS and 7.74 M and (ii) 20.00% BOFS and 8.90 M, which maximize the BOFS content with lower molarity and improve the mechanical properties with lower water absorption and porosity, respectively. The proposed methodology maximizes the utilization of waste BOFS in alkali-activated systems and may promote environmental and economic benefits.

## 1. Introduction

The utilization of waste materials has become crucial within the past decades due to challenges in minimizing waste disposal and preventing environmental degradation. Construction materials industries are regarded as the third-largest source of CO_2_ emissions among industrial sectors throughout the world and account for about 10% of global anthropogenic CO_2_ emissions [1]. Construction materials sectors are mostly related to concrete manufacturing, and significantly increasing construction activities have caused the production of cement to increase at an alarming rate. The fact that cement production releases approximately 8 to 10% of total CO_2_ [2] has prompted researchers across the world to explore substitutions for traditional cement or concrete with the purpose of minimizing the carbon footprint and reducing environmental concerns. The increasing demand for cement has led to the significant consumption of raw materials and CO_2_ emissions and thus requires environmentally friendly alternative materials as substitutes for cement.

Slags generated by metallurgical industries are classified on the basis of (i) the ironmaking process and (ii) the steelmaking process. The ironmaking process produces blast furnace slag, and, on the other hand, the steelmaking process results in the generation of basic oxygen furnace slag (BOFS), electric-arc furnace slag (EAFS), and ladle slag (LS) [3]. BOFS is one of the waste materials generated in considerable amounts during the steelmaking process [4,5]. Approximately 71.9% of steel was reported to be produced via an oxygen furnace [6], with a BOFS generation of about 100 to 200 kg per ton of steel production [7], exemplifying the considerable amount of BOFS generation. Moreover, about 15.7 million tons of steelmaking slag production was reported for 2018 in Europe, from which around 2.0 Mtons was reported to be disposed of as waste [8]. The disposed BOFS might be harmful to both the environment and living organisms since it releases toxic species during the processes of aging and the leaching of metallic compounds such as Fe_2_O_3_, Al_2_O_3_, and MgO present in BOFS [9]. Therefore, an alternative approach should be established to efficiently utilize generated BOFS waste.

Several studies on the utilization of BOFS in road construction, in asphalt, and as an aggregate have been reported in the literature. However, the expansion behavior of BOFS due to the existence of free CaO and MgO limits its application, which remains a challenge [10]. Several attempts have been made to stabilize free lime to minimize the expansion. Liu et al. [11] remelted and solidified raw BOFS under Ar and air atmosphere conditions and reported that BOFS processed in air significantly stabilized the free lime content and decreased the RO phases. Similarly, Morone et al. [12] claimed that a carbonation and granulation treatment might be a reliable technique to stabilize the volumetric instability of BOFS. In this methodology, BOFS was exposed to CO_2_ to allow the formation of CaCO_3_ via its reaction with free CaO present in BOFS particles. BOFS, having fineness similar to that of cement, can be used in small amounts as an additive in cement without undermining the integrity of the cement [13]. A study by Ma et al. [14] revealed that the compressive strength of the paste formulated with carbonated BOFS and cement was reduced with increasing BOFS content. Lin et al. [15] studied the synergetic valorization of BOFS and stone coal with the aim to recover metal and prepare glass ceramics. They obtained a final modified slag, which was successfully utilized to produce glass ceramics with a maximum bending strength of 95.83 MPa. Sun et al. [16] utilized BOFS aggregate to replace natural limestone in a metakaolin-based geopolymer. The metakaolin-based geopolymer concrete that completely replaced natural limestone yielded enhanced physical and mechanical properties.

Several studies concerning the application of BOFS have been reported in the literature. However, studies regarding the alkali activation of BOFS are still limited. Alkali-activated materials are synthesized using aluminosilicate materials and alkaline activators [17], which have gained significant attention from researchers. Alkali-activated materials exhibit remarkably superior mechanical durability properties or even outperform Portland cement [17,18]. Industrial by-products or mineral admixtures such as fly ash [19,20], ground granulated blast furnace slag (GGBFS) [21,22], metakaolin [23,24], and waste glass [25,26] are used to produce alkali-activated materials, whose major hydration component is silica-alumina gel [27,28].

A few studies have been reported on the utilization of BOFS either in alkaline activation [4,29] or as a cement replacement [14]. However, the use of an alkali-activated mortar (AAM) incorporating BOFS and GGBFS activated with an alkaline solution of sodium hydroxide (NaOH) remains unexplored. More attention has been paid to using steelmaking slags such as EAFS [30,31,32,33] and LS [34,35,36,37,38], while less attention has been paid to using other waste products such as BOFS for AAMs. Therefore, this study primarily aimed to propose an environmentally friendly method that efficiently utilizes BOFS generated as waste from steel industries to potentially promote environmental benefits and address economic problems related to waste BOFS. In the present study, the fresh, hardened, and microstructural properties of AAM consisting of different ratios of BOFS blended with GGBFS activated with various NaOH molarities have been investigated. The reason behind blending GGBFS with BOFS is the pozzolanic properties of GGBFS, which is involved in the reaction with precipitated Ca(OH)_2_ [32,39] produced due to free CaO present in BOFS, and this may stabilize the volume expansion [40]. Statistical interpretation and mathematical modeling were also performed using response surface methodology (RSM) to investigate the effect of the BOFS ratio and the concentration of NaOH on the investigated properties. RSM is a vital tool that involves analyzing and modeling the response of interest when it is influenced by several input parameters [41]. RSM offers an effective way to design experimental conditions and assess the relationship between independent and dependent variables with an aim to optimize and obtain targeted results [42]. The material consumption and cost can be significantly minimized once a reliable prediction model for the investigated properties of AAM is established.

## 2. Materials and Methodology

### 2.1. Raw Materials

**Figure 1:**
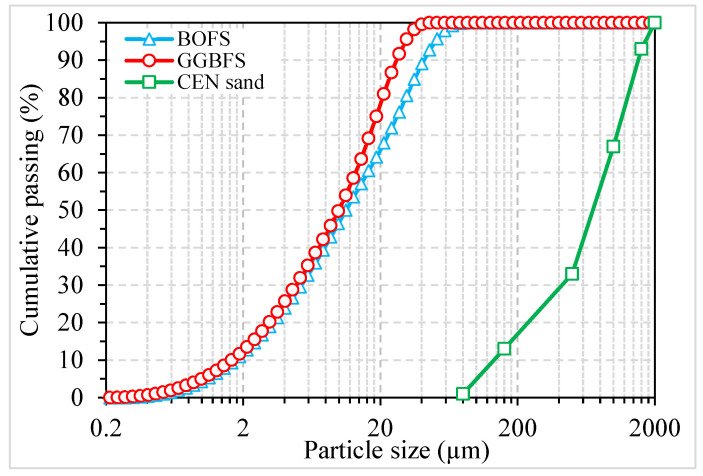
Particle size distribution of binders and sand.

**Figure 2:**
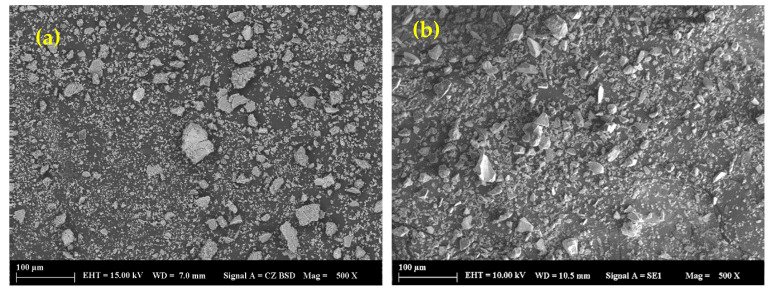
SEM photographs of (**a**) BOFS and (**b**) GGBFS.

**Figure 3:**
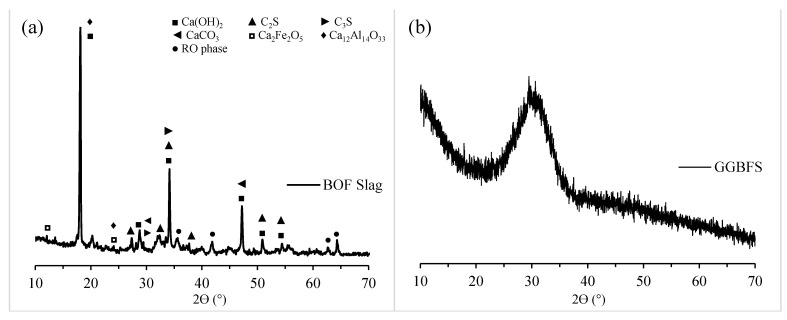
XRD of raw (**a**) BOFS and (**b**) GGBFS.

Basic oxygen furnace slag (BOFS) and ground granulated blast furnace slag (GGBFS), supplied by Erdemir Steel factory (Ereğli, Turkey) and Oyak Cement factory (Bolu, Turkey), respectively, were used to investigate the various properties of AAM. BOFS was initially dried and ground using mechanical disc grinder. Commercial NaOH with a pH value >14.0 and a molecular weight of 40.0 was used as the alkaline activator. CEN standard sand with particle sizes ranging between 0.08 and 2.00 mm was utilized as fine aggregate to manufacture mortar samples. Figure 1 shows the particle size distributions of BOFS, GGBFS, and sand. It can be observed that BOFS particles were slightly coarser compared to those of GGBFS since the d50 and d90 of BOFS were 11.2 and 41.4 µm, and those of GGBFS were 9.93 and 26.2 µm, respectively. The SEM images of the binders shown in Figure 2 indicate that BOFS has a rather rough surface texture compared to GGBFS, and both show a highly angular particle shape. The specific gravities of the ground BOFS, GGBFS, and sand were 3.01, 2.91, and 2.67, respectively. An X-ray fluorescence analysis was performed to determine the chemical composition of the binding materials, and the results are tabulated in Table 1. X-ray diffractometry analysis (XRD) was performed in the raw BOFS and GGBFS to determine their crystalline phases, and the results are shown in Figure 3. The majority of the crystalline phases present in BOFS were Ca(OH)_2_, C_2_S, C_3_S, Ca_2_Fe_2_O_5_, Ca_12_Al_14_O_33_, and RO phases [4]. Moreover, GGBFS had an amorphous structure (Figure 3b), showing a broader hump in the range between 25 and 40° 2θ, with crystalline phases such as SiO_2_, MgCO_3_, and CaCO_3_.

### 2.2. Design of Experiment, Model Efficacy Evaluation, and Mix Proportion

In this study, the commercially available Design-Expert^®^ 11 software was implemented to perform statistical interpretation, mathematical modeling, and the optimization of the mix designs using RSM. RSM is considered an efficacious statistical tool that is mainly employed for experimental design, mathematical modeling, and optimization [43]. RSM assists in the evaluation of responses that are affected by one or more factors [44]. Face-centered central composite design (FCCD), a subset of RSM, was used to statistically examine the effects of the independent parameters, namely, the BOF ratio and the molarity of NaOH, on the dependent variables: flow values, compressive strength, flexural strength, and water absorption. The influence of each parameter and the interaction among the variables were investigated using analysis of variance (ANOVA). A second-order regression model was used to determine the optimum condition of the investigated responses, as shown in the general formula in Equation (1) [45].

(1) $$Y=\beta_{o}+\sum_{i=1}^{k}\beta_{ii}X_{i}+{\left(\sum_{i=1}^{k}\beta_{ii}X_{i}\right)}^{2}+\sum_{i=1}^{k-1}\sum_{j=i+1}^{k}\beta_{ii}X_{i}X_{j}+Ѐ\;$$

where *Y* and *β* represent the predicted response and regression coefficient, respectively. *Xi* and *Xj* denote the coded terms of parameters, *k* denotes the number of parameters studied in the experiment, *i* and *j* are the linear coefficient and quadratic coefficient, respectively, and *ɛ* is the observed error.

**Figure 4:**
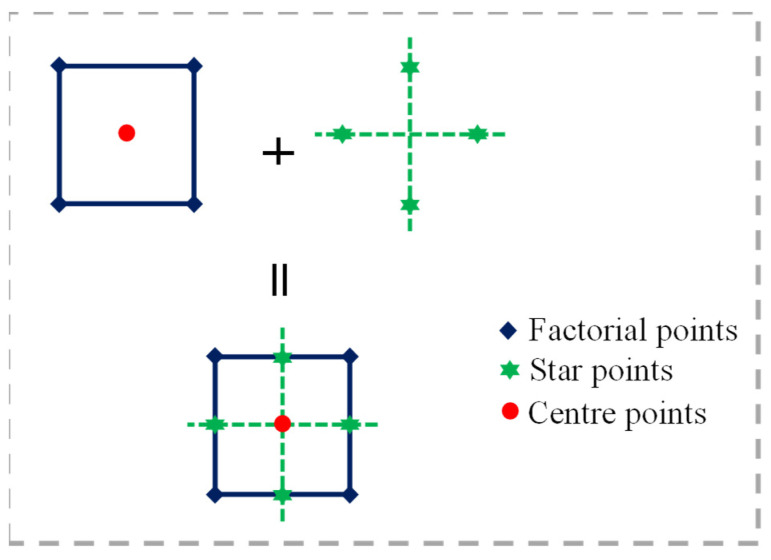
Visual representation of FCCD.

The values of the BOFS ratio by mass of binder and NaOH molarity varied in the ranges of 20–60% and 2–10 M, respectively. A visual representation of FCCD is shown in Figure 4, and Table 2 depicts the actual and coded terms of the input parameters. The BOFS ratio and NaOH molarity varied in three different levels, namely, axial or star points (±α) = 1, corner or factorial points (±1), and the center points. A total of 13 mix proportions, tabulated in Table 3, were obtained and consisted of 2 independent factors, 8 non-center points, and 5 replicates at the center points. Replicates at the center are very important since they assist in the estimation of the experimental error [45]. The water-to-binder ratio was kept constant at 0.4 for all mixes. Similarly, the volumes of sand and paste were used in equal amounts for each mix.

The assessment of the predicted *RSM* models was performed based on the mean square error (*MSE*), root-mean-square error (*RMSE*), and Nash–Sutcliffe coefficient efficiency (*NSE*), shown in Equations (2)–(5) [46,47]. These values were determined using experimental/observed values (*O_V_*s) and predicted values (*P_V_*s).

(2) $$MSE=\sum \frac{{\left(P_{V}-O_{V}\right)}^{2}}{N}$$

(3) $$RMSE=\sqrt{\sum \frac{{\left(P_{V}-O_{V}\right)}^{2}}{N}}=\sqrt{MSE}\;$$

(4) $$N_{t}=\left(\frac{SD}{RMSE}\right)-1\;$$

(5) $$NSE=1-{\left(\frac{1}{N_{t}+1}\right)}^{2}$$

where *N* is the sample size, *SD* represents the standard deviation of the observed values, and *N_t_* denotes the number of times the *SD* is greater than *RMSE*. The efficiency of the predicted *RSM* models can be categorized in terms of very good, good, acceptable, and satisfactory for *NSE* ≥ 0.90, 0.80–0.90, 0.65–0.80, and <0.65, respectively. In addition, the performance of a model can be categorized as very good, good, acceptable, and satisfactory if the *SD* value is in the ranges of ≥3.2*RMSE*, 2.2*RMSE*–3.2*RMSE*, 1.2*RMSE*–2.2*RMSE*, and <1.7*RMSE*, respectively [46].

### 2.3. Specimen Preparation

The mix proportions obtained from FCCD (Table 3) were used to manufacture the AAM specimens. The mix ID, for instance, designated by B0.2-6 denotes 20% BOFS content by mass of total binder and 6 M NaOH. The AAM mixes contained 50% sand by volume. The alkaline solution was prepared by mixing solid NaOH flakes with tap water in graduated cylinders, air-tightened, and left to cool under laboratory conditions prior to mixing. The blend of BOFS and GGBFS powder was initially mixed and introduced into a mixer bowl containing the alkaline solution, and thereafter, mixing was initiated. The sand was gradually added, and the mixing of the mortar continued to ensure a homogeneous mixture. The fresh mortar was used to determine the flow values and subsequently poured into molds for specified tests. The mortar was cast into 50 × 50 × 50 mm^3^ cubic molds to determine the compressive strength and water absorption and 40 × 40 × 160 mm^3^ prismatic molds to examine the flexural strength. The prepared samples along with the molds were covered with a waterproof plastic sheet and kept in laboratory conditions. The specimens were demolded after 24 h and conditioned in a humidity cabin having a relative humidity of 55–57% and a temperature of 20–22 °C until tested.

### 2.4. Laboratory Experimental Program

#### 2.4.1. Flow

The flow value of the fresh mortar was determined in accordance with ASTM C1437 to evaluate the workability. A minimum of four diameter readings were recorded to the nearest millimeter. The flow diameter of each mortar mix was calculated to the nearest 1% using Equation (6).

(6) $$Flow=\frac{\Phi }{\Phi_{0}}\times 100\%\;$$

where *Φ* is the difference between the average of four diameter readings and the original base diameter, and *Φ_o_* is the diameter of the original base.

#### 2.4.2. Compressive Strength

Compressive strength tests were performed on 50 × 50 × 50 mm^3^ cubic mortar samples at 28 days following ASTM C109. A minimum of three samples for each mortar mix were tested using a universal compression machine (Alşa, Istanbul, Turkey) with a loading rate of 900 to 1800 N/s at the specified age, and the average values were recorded.

#### 2.4.3. Flexural Strength

A flexural strength test of the mortar specimens was performed at 28 days in accordance with the ASTM C348 standard. The test was performed using a universal compression machine with a loading rate of 40 ± 5 N/s on 3 specimens for each mix, and the flexural strength was calculated using Equation (7).

(7) Flexural strength=0.0028×N 

where *N* is the average of maximum loads (in *N*).

#### 2.4.4. Water Absorption and Permeable Pore Volume

The water absorption and permeable pore volume were determined in accordance with ASTM C 642-13, with a slight modification to the pre-drying process of the mortar specimens. The mortar specimens were dried at 60 °C instead of 110 ± 5 °C to prevent excessive desiccation of the binding phases caused by thermal drying [48]. The specimens were placed in an oven at a temperature of 60 °C for 24 h. After removing the samples from the oven, they were allowed to cool in a desiccator to room temperature, and the mass (*A*) was measured. Subsequently, the samples were immersed in water for 48 h. After removing the specimens from the water, excess water was removed using a towel, and the saturated surface-dry mass (*B*) after immersion was recorded. The specimens were kept in boiling water for 5 h and allowed to cool down to room temperature. The soaked, boiled, and surface-dried masses (*C*) were measured, followed by the determination of the apparent mass (*D*) in water. The water absorption and permeable pore volume of the AAM specimens were determined by using Equations (8) and (9). Three specimens were used for each batch of mortar mixes, and the average values were recorded.

(8) $$Water\;absorption=\left(\frac{B-A}{A}\right)\times 100\%\;$$

(9) $$Permeable\;pore\;volume=\left(\frac{C-A}{C-D}\right)\times 100\%\;$$

#### 2.4.5. Microstructural Analysis

Alkali-activated paste specimens were prepared in plastic tubes with a volume of 50 mL to investigate the microstructural properties. The specimens in sealed tubes were maintained in laboratory conditions at 22 ± 2 °C. The samples were soaked in acetone to stop the hydration reactions at the test age and kept for analysis. A scanning electron microscopy (SEM) test was performed on slices cut from the selected paste samples at 28 days using a Zeiss EVO^®^ LS 10 SEM instrument (Carl Zeiss Microscopy GmbH, Jena, Germany) equipped with energy-dispersive X-ray spectroscopy (EDS) to analyze the surface morphology and determine reaction products.

An XRD analysis was performed on the raw precursors with a PANalytical X’Pert PRO diffractometer instrument (Malvern Panalytical Ltd., Malvern, United Kingdom) to distinguish the crystalline patterns in the powder precursor samples.

## 3. Results and Discussion

### 3.1. Fresh, Hardened, and Microstructural Properties

#### 3.1.1. Flow

**Figure 5:**
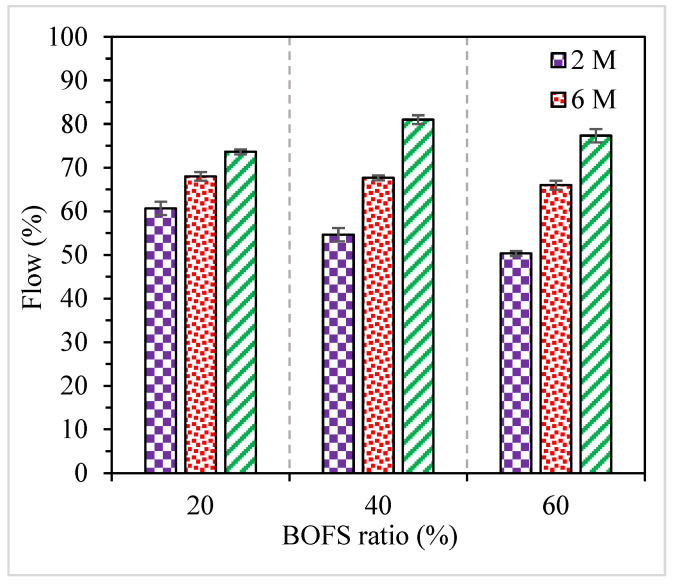
Flow values of AAM mixes (green is 10 M).

The variations in the flow of the fresh AAM mixes corresponding to their BOFS ratios and NaOH molarities are depicted in Figure 5, where the error bars indicate the standard deviation (SD). The flow values of the fresh mortar mix should be greater than 50%, which can be considered the minimum value for ease of molding [49]. The flow values of fresh AAM mixes varied in the range between 50 and 81%, and the fresh mortars were easily cast into the molds. The flow values of the AAM incorporating 20, 40, and 60% BOFS were obtained in ranges from 61 to 74%, 55 to 81%, and 50 to 77%, respectively. The lowest flow value was found in the mix incorporating 60% BOFS activated with 6 M NaOH, whereas the mortar mix containing 40% BOFS and 10 M NaOH corresponded to the highest flow value. It can be observed (Figure 5) that the increase in the molarity of NaOH consistently improved the flowability of the AAM mixes. The higher concentration of NaOH might have improved the dissolution of precursors, which subsequently resulted in more dissolved binders and hence enhanced the flow values [50]. Figure 5 shows that the effect of BOFS on the flow values of the mixes was inconsistent. In the case of 2 M NaOH, the flow was reduced from 61% to 50% when the BOFS ratio increased from 20% to 60%. On the other hand, with an increase in molarity, the flow remained constant or slightly increased as the BOFS ratio increased. The results indicate that the negative effect of BOFS on the flowability is compensated by a higher NaOH molarity, which improves the workability of the mixes.

#### 3.1.2. Compressive Strength

**Figure 6:**
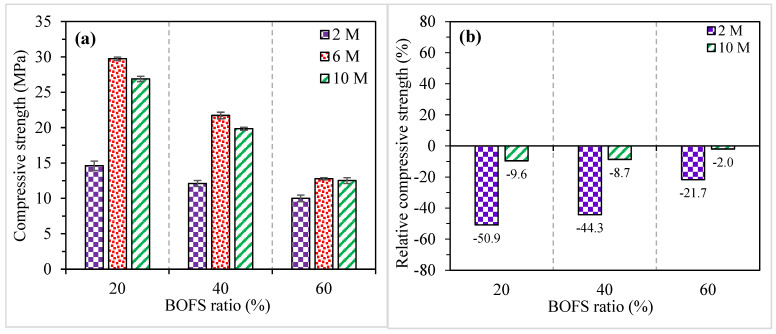
(**a**) Compressive strength and (**b**) relative compressive strength of specimens in comparison with 6 M.

Figure 6a shows the compressive strength results (the error bars indicate SD) and their variations with different BOFS ratios and NaOH molarities of the AAM samples at 28 days. The compressive strength of AAM specimens incorporating 20, 40, and 60% BOFS varied between 14.6 and 29.8 MPa, 12.1 and 21.7 MPa, and 10.0 and 12.8 MPa, respectively. It can be noticed that the compressive strength of the mortars consistently decreased with an increase in the BOFS ratio. Ismail et al. [48] also verified that the compressive strength of AAM containing GGBFS progressively decreased with the increasing GGBFS ratio. BOFS is generally recognized to be less reactive [51], and its higher inclusion level might have resulted in reduced compressive strength. In addition, the lower amounts of SiO_2_ and Al_2_O_3_ in BOFS might have resulted in the reduced formation of strength-giving reaction products when used in higher quantities.

On the other hand, the increase in NaOH molarity improved the compressive strength up to a certain limit, after which it decreased. The compressive strength of mortar specimens synthesized with 6 M NaOH outperformed both 2 and 10 M irrespective of the BOFS content. The reason behind the decrease in the compressive strength of samples containing 10 M NaOH might be due to the higher NaOH concentration, which might have hindered the reaction process due to the existence of surplus hydroxide ions and consequently reduced aluminosilicate gel precipitation [52]. Another reason might be the lack of silicates [53] required to equivalently react with excess hydroxide ions for the same group of samples.

Figure 6b compares the relative compressive strength of the AAM mixes with 2 and 10 M NaOH to that of the mixes activated with 6 M NaOH. The AAM mixes containing 20, 40, and 60% BOFS had 50.9, 44.3, and 21.7% lower compressive strength compared to the mixes with 6 M NaOH, respectively. Similarly, the compressive strength slightly decreased by 9.6, 8.7, and 2.0% in the AAM mixes with 10 M containing 20, 40, and 60% BOFS ratios, respectively, compared to the mortar mixes activated with 6 M NaOH. It can be further noticed that the reduction in compressive strength in 2 and 10 M AAM is lower in the mixes with higher contents of BOFS.

#### 3.1.3. Flexural Strength

**Figure 7:**
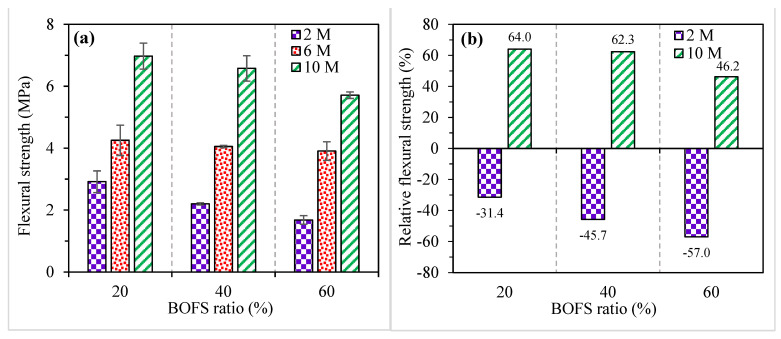
(**a**) Flexural strength and (**b**) relative flexural strength of specimens in comparison with 6 M.

The flexural strength results of the AAM specimens with various replacement ratios of BOFS and NaOH molarities at 28 days are depicted in Figure 7a (the error bars indicate SD). The flexural strength of AAM samples consistently decreased upon the addition of BOFS (reduction in GGBFS), which is consistent with the compressive strength results. The flexural strength for the AAM mixes containing 20, 40, and 60% BOFS varied between 2.9 and 7.0 MPa, 2.2 and 6.6 MPa, and 1.7 and 5.7 MPa, respectively. The maximum flexural strength (7.0 MPa) was achieved in the AAM mix incorporating 20% BOFS with 10 M NaOH, whereas the mix with 60% BOFS and 2 M NaOH had the minimum flexural strength (1.7 MPa). From the results, it is worth noting that the flexural strength of the AAM samples significantly improved with the increase in NaOH molarity, which provided a denser microstructure, as verified by the SEM micrographs.

The relative flexural strength of AAM samples containing 2 and 10 M ranged between −31.4% and −57.4% and between 64.0% and 46.2% respectively, in comparison with the specimens activated with 6 M NaOH, as shown in Figure 7b.

#### 3.1.4. Water Absorption and Volume of Permeable pores

**Figure 8:**
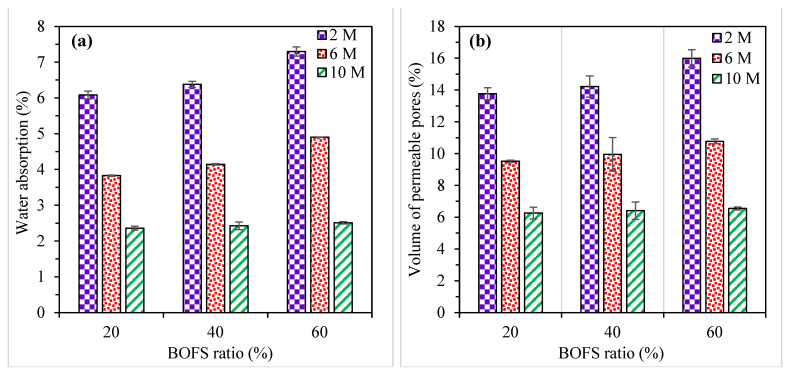
(**a**) The variation in the water absorption and (**b**) volume of permeable pores of AAM specimens.

The water absorption and volume of permeable pores of AAM mixes are illustrated in Figure 8, where the error bars indicate SD. Figure 8a shows that the inclusion of BOFS moderately increased the water absorption. This observation can be attributed to the reduced GGBFS content. GGBFS has a slightly finer particle size (see Figure 1) than BOFS, which might have assisted in filling the pores and consequently resulted in lower water absorption [54]. On the contrary, the increase in NaOH molarity significantly reduced the water absorption at all replacement ratios of BOFS. The water absorption of mortar mixes with lower NaOH molarity (2 M) was in the range between 6.1 and 7.3%, whereas the samples activated with 10 M NaOH varied between 2.4 and 2.5%. The higher water absorption values of mortar mixes with a lower alkaline concentration can be attributed to the higher volume of permeable pores and vice versa. Similar observations were also reported by [55,56], in which the increase in the concentration of the NaOH activator decreased the water absorption values.

The increment in NaOH molarity induced a significant reduction in the volume of permeable pores, whereas this value slightly increased with the increase in the BOFS ratio (Figure 8b). The trend observed in the volume of permeable pores was similar to that in the water absorption of the AAM specimens. The volume of permeable pores varied between 6.3 and 13.8%, 6.4 and 14.2%, and 6.6 and 16.0% for the AAM samples containing 20, 40, and 60% BOFS, respectively. For the mortar specimens activated with 2, 6, and 10 M, the volume of permeable pores ranged between 13.8 and 16.0%, 9.5 and 10.8%, and 6.3 and 6.6%, respectively. The volume of permeable pores was the lowest (6.3%) in the mortar specimens formulated with 20% BOFS and 10 M, and it was the highest (16.0%) in samples composed of 60% BOFS activated with 2 M NaOH.

#### 3.1.5. SEM-EDS Analysis

**Figure 9:**
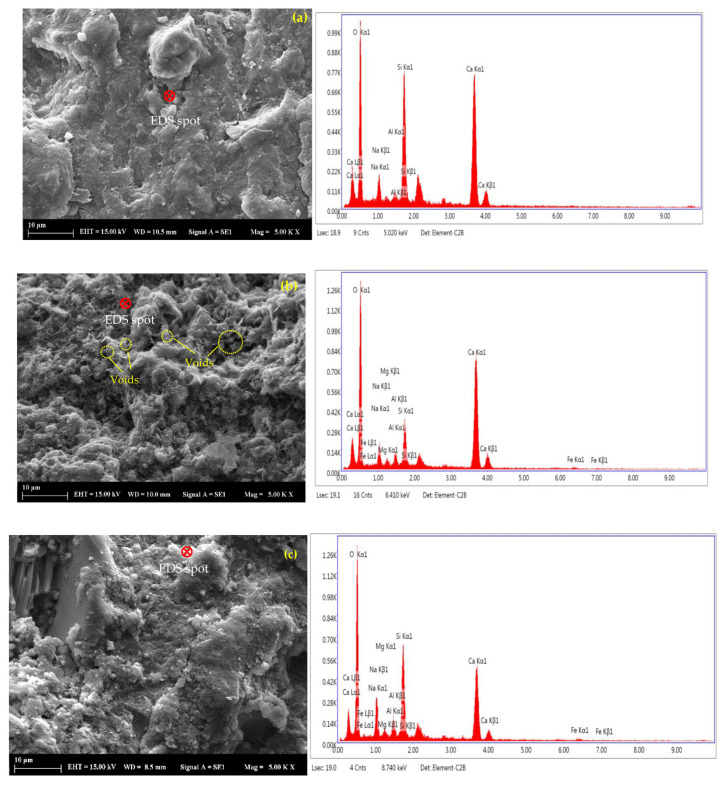
SEM-EDS of alkali-activated pastes: (**a**) B0.2-6, (**b**) B0.6-2, and (**c**) B0.6-6.

SEM imaging was conducted on the corresponding selected alkali-activated pastes of B0.2-6, B0.6-2, and B0.6-6 at 28 days to study the effect of the BOFS ratio and NaOH molarity on the microstructural features. The SEM micrographs along with the EDS results of the paste samples are shown in Figure 9. The EDS analysis showed the abundant presence of Si, Ca, and Na elements, as well as Mg and Fe elements in lower amounts, which may indicate the formation of hydrated reaction products such as C-A-S-H and N-A-S-H. The SEM images show that B0.6-2 had a distinctive morphology compared to B0.6-6, and B0.6-2 exhibited a more porous structure than B0.6-6, which might have contributed to the increase in water absorption and permeable pore volume. Figure 9a,c reveals that for the same NaOH molarity, a lower BOFS ratio yielded a denser microstructure. The same was also noticed when comparing Figure 9b,c, where an increase in NaOH molarity at a constant BOFS ratio reduced the voids. These observations are in line with the mechanical test results, as an increase in NaOH molarity and an increase in the GGBFS ratio generally improved the compressive and flexural strengths.

### 3.2. Analysis of Variance and Regression Model Equations

The experimental results of flow, compressive strength, flexural strength, and water absorption were analyzed using RSM to determine the effects of the BOFS replacement ratio and NaOH molarity. The ANOVA results for the investigated properties are presented in Table 4. The coefficient of determination (R^2^) values were considered to examine the precision of the predicted quadratic models. A greater R^2^ (close to unity) denotes a desirable and reasonable relationship between the predicted and actual values [57]. It can be noticed that the R^2^ values of the analyzed responses were 0.95, indicating efficient predictive models. The R^2^ values for flow, compressive strength, flexural strength, and water absorption were found to be 0.97, 0.95, 0.95, and 0.99, respectively. Furthermore, the significance of the model terms was evaluated using the probability (*p*-value) at a 95% confidence interval level. A *p*-value lower than 0.05 shows that the model or model terms are statistically significant. The *p*-values for flow, compressive strength, flexural strength, and water absorption models were calculated as <0.0001, 0.0002, 0.0002, and <0.0001, respectively, which indicated that all models were statistically significant. The model terms B and AB were significant for flow values, whereas all model terms (A, B, AB, and B^2^) were statistically significant, except quadratic term A for compressive strength. Similarly, only linear terms A and B were significant for flexural strength, while for water absorption, all model terms (both linear and quadratic) were significant.

In addition, the ratio of Fischer variation (F-value), which measures the variation in the data about its mean value, was also considered to validate the obtained response models. The higher the F-value is than 1.00, the more reliable the model [58]. The F-values for flow, compressive strength, flexural strength, and water absorption were 43.26, 25.76, 28.70, and 458.72, respectively. Even though B and AB were statistically significant, the model’s higher R^2^ value of 0.97, lower *p*-value < 0.0001, higher F-value of 43.26, and insignificant lack of fit (LOF) statistically validated the flow model. It is worth noting that the *p*-value (0.0012) of LOF is significant for water absorption, but it did not invalidate the analyzed model since the R^2^ value was about 0.99, implying that 99% of the total variability was well explained by the predicted model. The greater R^2^ values, lower *p*-values, higher F-values, and insignificant LOF (except water absorption) described the adequacy of the predicted quadratic models for all investigated properties.

Furthermore, the adequate precision (AP) values for flow, compressive strength, flexural strength, and water absorption were found to be 22.33, 15.82, 17.46, and 67.46, respectively. The AP values for all responses were greater than 4, which is desirable and supports that the developed quadratic models can be efficiently utilized for navigating the defined design space by FCCD. The actual regression equations for all investigated responses, in terms of both significant and insignificant influencing terms, are expressed by quadratic Equations (10)–(13).

(10) $$Flow=+60.8630-0.1921A+0.9226B+0.0438AB-0.0018A^{2}+0.0076B^{2}\;\;\;\;\;$$

(11) $$Compressive\;strength=+7.4843-0.0759A+6.2761B-0.0305AB-0.0005A^{2}-0.3429B^{2}\;\;\;\;\;\;\;\;\;\;\;\;\;$$

(12) $$Flexural\;strength=+2.7718-0.0405A+0.3670B-0.0001AB+0.0002A^{2}+0.0129B^{2}$$

(13) $$Water\;absorption=+6.9300+0.0020A-0.5587B-0.0034AB+0.0005A^{2}+0.0146B^{2}\;\;\;\;\;\;\;\;\;\;\;\;\;\;\;\;\;\;\;\;\;\;$$

where *A* and *B* represent the BOFS ratio and NaOH molarity, respectively.

### 3.3. Perturbation and Normal Probability Plots

**Figure 10:**
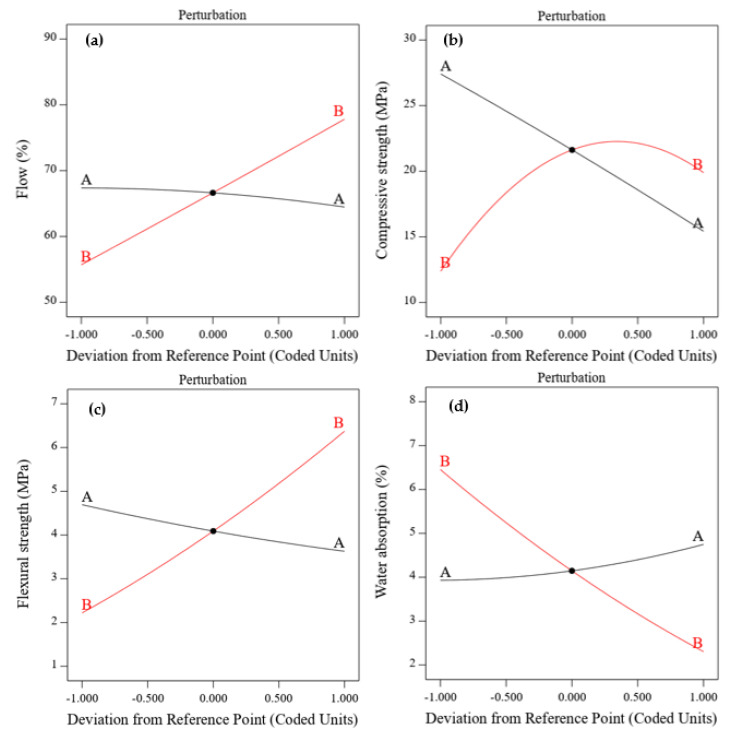
Perturbation plots of (**a**) flow, (**b**) compressive strength, (**c**) flexural strength, and (**d**) water absorption.

The perturbation plot explains the influence of independent variables on the response at a particular point [59]. The BOFS ratio formed a slight curvature, showing its low sensitivity to the flow and water absorption properties, as shown in Figure 10a,d, respectively. The observation from the perturbation plot (Figure 10b) implies that the effect of NaOH molarity was more sensitive to the compressive strength due to the formation of a sharp curvature compared to that of BOFS content. The formation of this sharp curvature indicates that NaOH molarity enhances the compressive strength up to a certain molarity.

**Figure 11:**
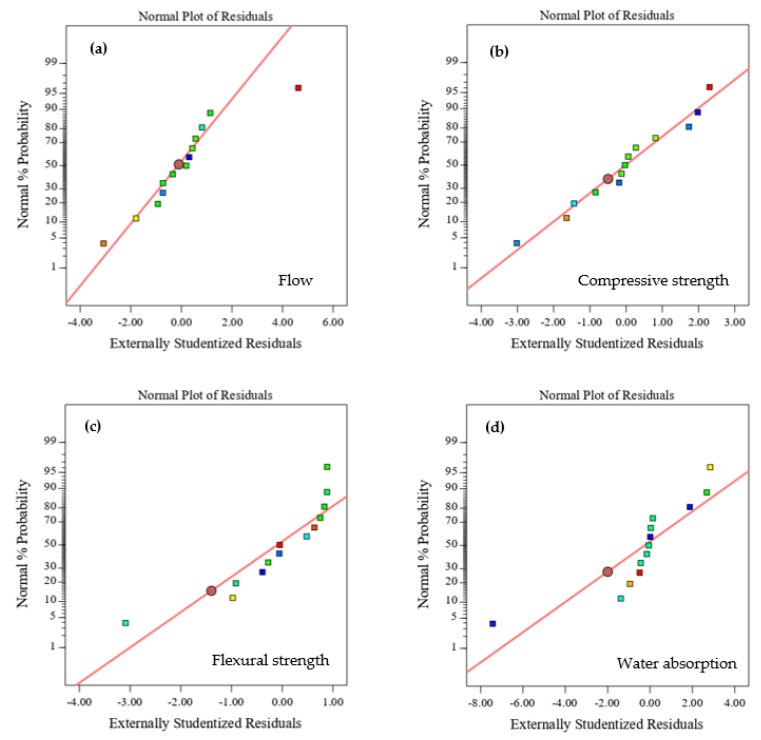
Normal probability plots of (**a**) flow, (**b**) compressive strength, (**c**) flexural strength, and (**d**) water absorption.

However, both the BOFS ratio and NaOH molarity seemed to form linear straight lines (Figure 10c) for flexural strength. Figure 11 presents the normal plots of the residual values for all responses, which aided in defining the appropriateness of the model. Figure 11a–c show that the flow values, compressive strength, and flexural strength residual plots formed straight lines, which satisfied the plot of the studentized residual against the normal percentage of probability; the exception was water absorption, which showed comparatively scattered data points (Figure 11d).

### 3.4. Predicted vs. Actual Plots

**Figure 12:**
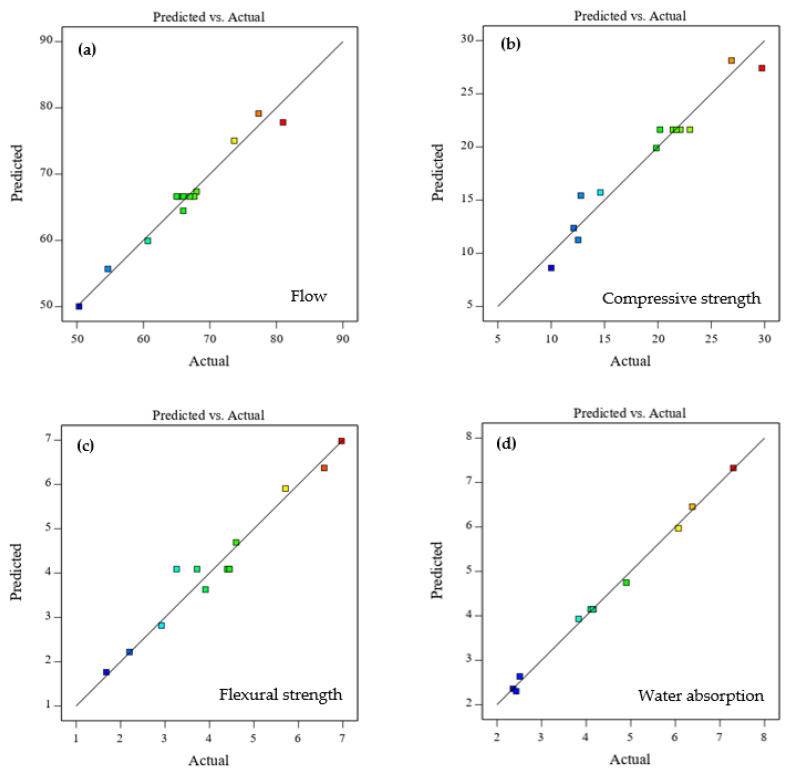
Predicted vs. actual plots for (**a**) flow, (**b**) compressive strength, (**c**) flexural strength, and (**d**) water absorption.

The predicted values compared to the actual values of the investigated properties are shown in Figure 12. The predicted values were very close to the actual values of flow, compressive strength, flexural strength, and water absorption. However, a few points were not on the line for compressive and flexural strengths, as shown in Figure 12b,c. On the other hand, the consistency of the predicted and experimental flow values was higher compared to compressive and flexural strengths since very few points were dispersed far from the line, as confirmed in Figure 12a. The predicted values of water absorption (Figure 12d) were found to be more consistent with and closer to the experimental values compared to the other responses, which can also be confirmed by the maximum R^2^ value of 0.99. Nevertheless, the predicted and experimental values followed linear trends for each response and thus verified the reliability and prediction of the response models.

### 3.5. Contour and 3D Response Surface Plots of the Responses

**Figure 13:**
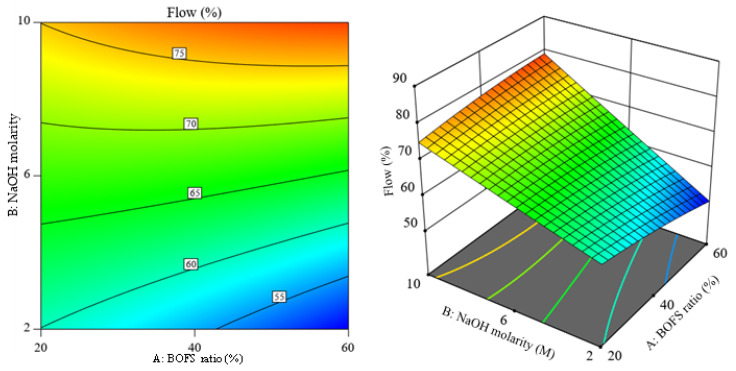
Contour plot and 3D response surface of flow values.

**Figure 14:**
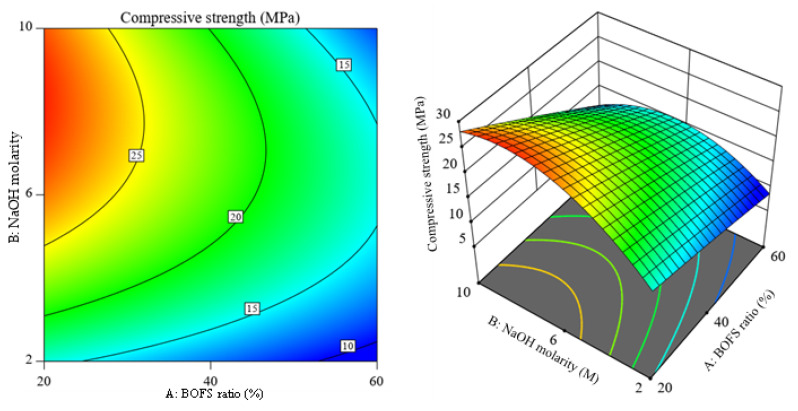
Contour plot and 3D response surface of 28-day compressive strength.

**Figure 15:**
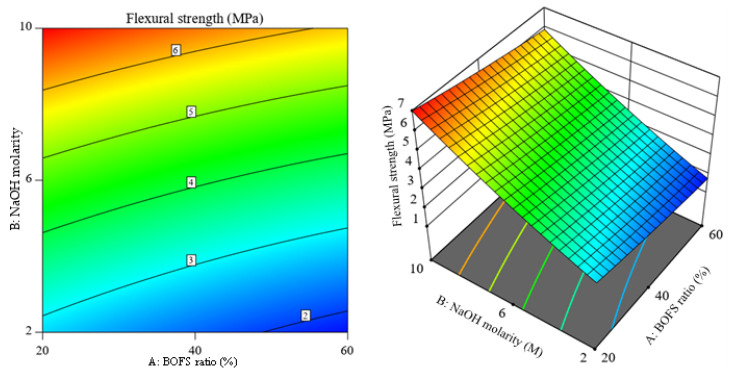
Contour plot and 3D response surface of 28-day flexural strength.

**Figure 16:**
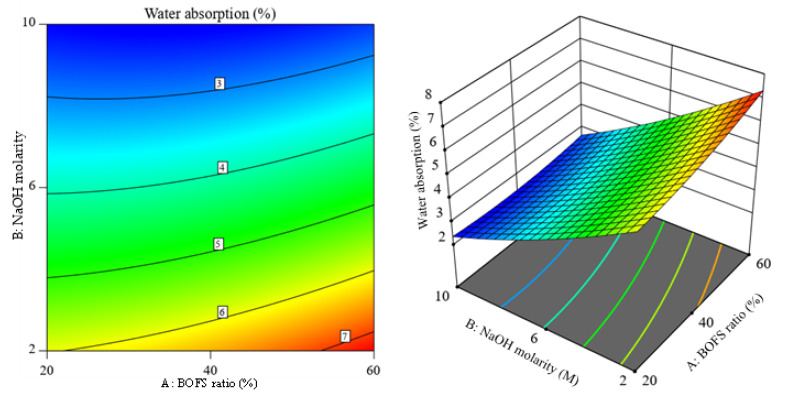
Contour plot and 3D response surface of water absorption.

Figure 13 illustrates the 3D response surface and its corresponding contour plot for the response flow of the AAM samples, and it shows a slight curvature with the BOFS ratio. The alteration of NaOH molarity was more sensitive to the flowability for the selected range of BOFS content. The distorted contours observed in Figure 14 imply that there is less interaction between independent variables. However, the curvature seen along the molarity axis denotes the possible optimal value for NaOH molarity. The curvature of the 3D response surface graph shown in Figure 15 depicts that the NaOH molarity more markedly influenced the flexural strength compared to the BOFS ratio. Figure 16 visualizes the variation in the response surface and contour plots with the BOFS ratio and NaOH molarity, in which the contours are slightly curved, denoting comparatively fewer interactions between the independent variables for water absorption. The bluish section (Figure 16) provides the preferred water absorption values for the studied mortar specimens.

### 3.6. Predictive Performance of the Derived RSM Models

The predictive efficiency of the models obtained by RSM defined by FCCD was classified based on NSE, SD, and RMSE values, and the results are tabulated in Table 5. In terms of NSE criteria, the RSM models were categorized as very good for predicting flow, compressive strength, flexural strength, and water absorption since their corresponding NSE values were greater than 0.90. Water absorption had the maximum NSE value (1.00), followed by flow (0.97), flexural strength (0.96), and compressive strength (0.95). It has been previously reported that higher efficiency and goodness of fit are directly related to lower MSE and RMSE [60]. The lower MSE and RMSE values for water absorption imply the appropriate efficiency of the predicted RSM models, which is also consistent with the corresponding NSE value of 1.00. Furthermore, all responses were categorized as very good based on the SD and RMSE specifications, as the corresponding SD values of the responses were greater than 3.2RMSE. The obtained results and model efficiency classification clearly suggest that the predicted RSM models can be accurately used to navigate the defined design space to estimate the flow, compressive strength, flexural strength, and water absorption of the AAM samples.

### 3.7. Optimization and Experimental Validation

In this part of the study, numerical optimization was performed using the multi-objective optimization technique, as this method assists in optimizing several responses concurrently [61,62]. The optimal values of the independent parameters (A: BOFS ratio; B: NaOH molarity) were determined by setting the optimization targets, namely, optimization-1, in which the goals of all parameters were within the range, and optimization-2, where compressive strength and flexural strength were maximized, water absorption was minimized, and the remaining parameter goals were within the range. The optimal values of BOFS and NaOH molarity in optimization-1 were found to be 24.61% and 7.74 M, respectively, whereas BOFS content and NaOH molarity were calculated as 20.00% and 8.90 M, respectively, in optimization-2, and their corresponding values of dependent variables are illustrated in Table 6. The outcome of multi-objective optimization was assessed by the desirability value (*dj*), which can be computed using the geometrical mean of each response’s desirability, as expressed in Equation (14) [63].

(14) D=(d1r1×d1r1×……×dnrn)1n

where *ri* and *n* represent the importance level for each objective function *di* and the total number of responses considered, respectively.

**Figure 17:**
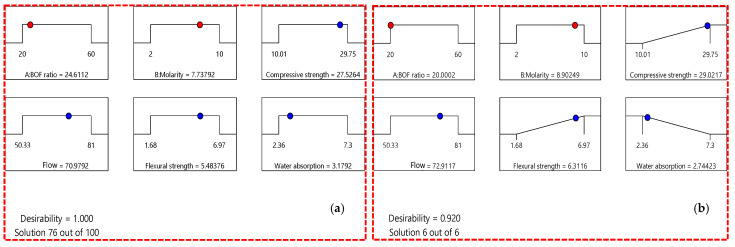
Ramp diagram of multi-objective optimization for (**a**) optimization-1 and (**b**) optimization-2.

The dj value ranges between 0 and 1, where 1 represents the ideal response and 0 denotes an undesirable response. The desirability of optimization-1 and optimization-2 was found to be 1.00 and 0.920, respectively. The predicted optimized mix proportions of optimization-1 and optimization-2 were experimentally investigated three times, and the average values were noted to validate the appropriateness of the response models and optimization results. In addition, the error between the predicted and experimental results was calculated by using Equation (15). The results of the optimization study are tabulated in Table 6. Similarly, the graphical representation of the independent factors and the responses are presented through the optimization ramp shown in Figure 17. In both optimization-1 and optimization-2, the experimental values were comparable to the predicted values, with errors ranging between 0.57 and 1.43%, 0.87 and 1.56%, 2.05 and 0.31%, and 2.58 and 6.20% for flow, compressive strength, flexural strength, and water absorption, respectively.

In optimization-1, the BOFS content was higher, and NaOH molarity was lower; however, these values were reversed in optimization-2. On the other hand, optimization-1 can be considered efficient in terms of a higher amount of BOFS utilization with a lower dosage of the activator, whereas optimization-2 can be appropriate to achieve higher flow, greater compressive and flexural strengths, and lower water absorption. Considering the optimization study, the predicted response models were appropriate and suitable to navigate the defined design space since the experimental and predicted results were well correlated and thus validated the obtained response models.

(15) $$Error\;\left(\%\right)=\left|1-\frac{Predicted\;value}{Experimental\;value}\right|\times 100\%\;$$

## 4. Conclusions

The present study aimed to propose the efficient utilization of BOFS generated as waste to potentially promote environmental benefits. The properties of mortar mixes synthesized with different ratios of BOFS and GGBFS activated with NaOH were investigated. RSM was employed to statistically interpret and optimize independent and dependent variables. The following conclusions are outlined based on the experimental and statistical study:

- The compressive strength of AAM samples activated with 6 M NaOH reached about 30 MPa and was superior to those activated using 2 and 10 M. In addition, increasing the BOFS content consistently decreased the compressive strength and flexural strength of AAM samples.
- The water absorption and permeable pore volumes of AAM samples significantly decreased with an increase in NaOH molarity, whereas they slightly increased with an increase in the BOFS ratio.
- The SEM observations revealed that increasing the NaOH molarity and reducing the BOFS ratio resulted in a denser microstructure, which is in agreement with the physical and mechanical test results.
- The ANOVA results revealed that the obtained response models were accurate and statistically significant. The proposed quadratic models can be appropriately used to predict the response by navigating the defined design space by FCCD.
- The optimal mix proportions of BOFS and NaOH molarity were found to be 24.61% and 7.74 M (optimization-1) for the efficient utilization of BOFS with a lower NaOH concentration. In addition, the optimal mix design of 20.00% BOFS and 8.90 M NaOH (optimization-2) performed better in achieving higher flow, greater compressive and flexural strengths, and lower water absorption.
- The proposed methodology can promote environmental benefits by utilizing BOFS to produce alkali-activated mortars. This method may also address the economic and environmental issues due to the disposal of BOFS. Furthermore, this study might also create awareness among steel manufacturers that are involved in BOFS generation by visualizing its commercial importance in construction.

## Figures and Tables

**Table 1:** Oxide compositions of BOFS and GGBFS.

Precursor	Component (wt%)												
	SiO_2_	Al_2_O_3_	Fe_2_O_3_	CaO	MgO	SO_3_	K_2_O	Na_2_O	TiO_2_	P_2_O_5_	Cr_2_O_3_	Mn_2_O_3_	LOI *
BOFS	8.8	4.27	23.28	41.07	5.25	1.74	0.01	0.1	0.2	0.65	0.15	2.53	13.12
GGBFS	39	12.5	1	37.5	5	0.3	0.2	0.6	-	-	-	-	0.02

*: Loss on ignition (LOI).

**Table 2:** Face-centered central composite design for 2 factors at 3 levels.

Levels	Factor 1 A: BOF Ratio (wt%)	Factor 2 B: NaOH Molarity (M)
−1	20	2
0	40	6
+1	60	10

**Table 3:** Details of experimental mix design.

Mixture ID	Coded		Actual		Mix Proportion		
	A	B	A (wt%)	B (M)	BOFS (wt%)	GGBFS (wt%)	NaOH Molarity (M)
B0.2-2	−1	−1	20	2	20	80	2
B0.4-2	0	−1	40	2	40	60	2
B0.6-2	1	−1	60	2	60	40	2
B0.2-6	−1	0	20	6	20	80	6
B0.4-6 *	0	0	40	6	40	60	6
	0	0	40	6	40	60	6
	0	0	40	6	40	60	6
	0	0	40	6	40	60	6
	0	0	40	6	40	60	6
B0.6-6	1	0	60	6	60	40	6
B0.2-10	−1	1	20	10	20	80	10
B0.4-10	0	1	40	10	40	60	10
B0.6-10	1	1	60	10	60	40	10

A: BOFS. B: NaOH molarity. * Represents identical mix designs (5 center points).

**Table 4:** ANOVA results of the analyzed responses.

Response	Source	SS	DF	MS	F-Value	*p*-Value	
Flow	Model	796.31	5	159.26	43.26	<0.0001 *	SD = 1.92 R^2^ = 0.97 AP = 22.33
	A: BOFS ratio	12.56	1	12.56	3.41	0.1073	
	B: Molarity	733.28	1	733.28	199.18	<0.0001 *	
	AB	49.00	1	49.00	13.31	0.0082 *	
	A²	1.41	1	1.41	0.38	0.5562	
	B²	0.04	1	0.04	0.01	0.9191	
	Residual	25.77	7	3.68			
	Lack of fit	20.73	3	6.91	5.49	0.0668	
	Pure Error	5.038	4	1.259			
	Cor. Total	822.08	12				
Compressive strength	Model	424.03	5	84.81	25.76	0.0002 *	SD = 1.81 R^2^ = 0.95 AP = 15.82
	A: BOFS ratio	215.28	1	215.28	65.39	0.0001 *	
	B: Molarity	84.75	1	84.75	25.74	0.0014 *	
	AB	23.86	1	23.86	7.25	0.0310 *	
	A²	0.11	1	0.11	0.03	0.8584	
	B²	83.16	1	83.16	25.26	0.0015 *	
	Residual	23.04	7	3.29			
	Lack of fit	18.85	3	6.28	6.00	0.0581	
	Pure Error	4.190	4	1.047			
	Cor. Total	447.07	12				
Flexural strength	Model	27.76	5	5.55	28.70	0.0002 *	SD = 0.44 R^2^ = 0.95 AP = 17.46
	A: BOFS ratio	1.70	1	1.70	8.77	0.0211 *	
	B: Molarity	25.88	1	25.88	133.74	<0.0001 *	
	AB	0.00	1	0.00	0.00	0.9825	
	A²	0.01	1	0.01	0.07	0.7957	
	B²	0.12	1	0.12	0.61	0.4614	
	Residual	1.35	7	0.19			
	Lack of fit	0.19	3	0.06	0.22	0.8757	
	Pure Error	1.160	4	0.290			
	Cor. Total	29.12	12				
Water absorption	Model	27.53	5	5.51	458.72	<0.0001 *	SD = 0.11 R^2^ = 0.99 AP = 67.46
	A: BOFS ratio	1.00	1	1.00	83.34	<0.0001 *	
	B: Molarity	25.83	1	25.83	2152.05	<0.0001 *	
	AB	0.29	1	0.29	24.29	0.0017 *	
	A²	0.10	1	0.10	8.59	0.0220 *	
	B²	0.15	1	0.15	12.52	0.0095 *	
	Residual	0.08	7	0.01			
	Lack of fit	0.08	3	0.03	51.52	0.0012 *	
	Pure Error	0.002	4	0.001			
	Cor. Total	27.62	12				

*: Significant; SS: summation of squares; Cor. Total: corrected total summation of squares; DF: degree of freedom; MS: mean square; AP: adequate precision.

**Table 5:** The goodness-of-fit assessment of response models.

Response	SD	MSE	RMSE	NSE	Nt	Outcome
Flow	8.28	1.98	1.41	0.97	4.88	Very good
Compressive strength	6.10	1.77	1.33	0.95	3.58	Very good
Flexural strength	1.56	0.10	0.32	0.96	3.83	Very good
Water absorption	1.52	0.01	0.08	1.00	17.87	Very good

**Table 6:** Multi-objective optimization of the mix design and response.

	Dependent and Independent Factors	Optimization Goal	Desirability	Predicted Values	Experimental Values	SD	Error (%)
Optimization-1	A: BOFS ratio (%)	In range	1.000	24.61			
	B: Molarity (M)	In range		7.74			
	Flow	In range		70.97	72.00	1.41	1.43
	Compressive strength	In range		27.53	27.77	1.54	0.87
	Flexural strength	In range		5.48	5.37	0.05	2.05
	Water absorption	In range		3.18	3.10	3.18	2.58
Optimization-2	A: BOFS ratio (%)	In range	0.920	20.00			
	B: Molarity (M)	In range		8.90			
	Flow	In range		72.91	73.00	1.29	0.57
	Compressive strength	Maximize		29.02	29.48	2.04	1.56
	Flexural strength	Maximize		6.31	6.33	0.50	0.31
	Water absorption	Minimize		2.74	2.58	0.10	6.20

## Data Availability

Not applicable.
